# A Molecular Genetic Timescale for the Diversification of Autotrophic Stramenopiles (Ochrophyta): Substantive Underestimation of Putative Fossil Ages

**DOI:** 10.1371/journal.pone.0012759

**Published:** 2010-09-16

**Authors:** Joseph W. Brown, Ulf Sorhannus

**Affiliations:** 1 Museum of Zoology and Department of Ecology & Evolutionary Biology, University of Michigan, Ann Arbor, Michigan, United States of America; 2 Department of Biology & Health Services, Edinboro University of Pennsylvania, Edinboro, Pennsylvania, United States of America; Natural History Museum of Denmark, Denmark

## Abstract

**Background:**

Stramenopiles constitute a large and diverse eukaryotic clade that is currently poorly characterized from both phylogenetic and temporal perspectives at deeper taxonomic levels. To better understand this group, and in particular the photosynthetic stramenopiles (Ochrophyta), we analyzed sequence data from 135 taxa representing most major lineages. Our analytical approach utilized several recently developed methods that more realistically model the temporal evolutionary process.

**Methodology/Principal Findings:**

Phylogenetic reconstruction employed a Bayesian joint rate- and pattern-heterogeneity model to reconstruct the evolutionary history of these taxa. Inferred phylogenetic resolution was generally high at all taxonomic levels, sister-class relationships in particular receiving good statistical support. A signal for heterotachy was detected in clustered portions of the tree, although this does not seem to have had a major influence on topological inference. Divergence time estimates, assuming a lognormally-distributed relaxed molecular clock while accommodating topological uncertainty, were broadly congruent over alternative temporal prior distributions. These data suggest that Ochrophyta originated near the Proterozoic-Phanerozoic boundary, diverging from their sister-taxon Oomycota. The evolution of the major ochrophyte lineages appears to have proceeded gradually thereafter, with most lineages coming into existence by ∼200 million years ago.

**Conclusions/Significance:**

The evolutionary timescale of the autotrophic stramenopiles reconstructed here is generally older than previously inferred from molecular clocks. However, this more ancient timescale nevertheless casts serious doubt on the taxonomic validity of putative xanthophyte/phaeophyte fossils from the Proterozoic, which predate by as much as a half billion years or more the age suggested by our molecular genetic data. If these fossils truly represent crown stramenopile lineages, then this would imply that molecular rate evolution in this group proceeds in a fashion that is fundamentally incompatible with the relaxed molecular clock model employed here. A more likely scenario is that there is considerable convergent morphological evolution within Heterokonta, and that these fossils have been taxonomically misdiagnosed.

## Introduction

The photosynthetic stramenopiles (Ochrophyta) [1] constitute a highly diverse clade within Heterokonta, a clade that also includes a number of heterotrophic lineages such as plant molds and aquatic pseudofungi [e.g. 2], [3]. The majority of published molecular phylogenetic analyses have indicated that the photosynthetic and non-photosynthetic stramenopiles form a monophyletic taxon [2], [3], [4], [5], [6], [7], [8]. Heterokonts are typically characterized by the presence of a flagellum with tripartite tubular hairs (stramenopiles) and a smooth flagellum (i.e. lacking mastigonemes), although these are secondarily reduced or lost in some lineages [8]. The closest living relative of the heterokont eukaryotes has traditionally remained unclear. However, in a number of recent studies, Rhizaria has been identified with high support values as the sister-lineage ([5], [6]; however, see [7], [9] for different inferred relationships).

The major ochrophyte lineages, often considered as different classes, include Aurearenophyceae, Bacillariophyceae, Eustigmatophyceae, Dictyochophyceae, Synchromophyceae, Chrysophyceae, Chrysomerophyceae, Bolidophyceae, Xanthophyceae, Synurophyceae, Schizocladiophyceae, Raphidophyceae, Pinguiophyceae, Phaeothamniophyceae, Phaeophyceae, Picophagea and Pelagophyceae. Due to inferred paraphyly, the status of Picophagea [3] as a distinct class has been questioned [10], [11]. Phylogenetic relationships among the major pigmented heterokont lineages are generally poorly resolved ([2], [12], [13]; see [8] for improved resolution). However, the majority of molecular systematic studies indicate that Oomycota is either the sole outgroup of the photosynthetic stramenopiles or that this taxon is part of a larger heterotrophic stramenopile lineage that constitutes the closest living relative of Ochrophyta [e.g. 2], [3], [8], [10], [11], [14].

The earliest fossil remains (*Palaeovaucheria*; Xanthophyceae) suggest that the photosynthetic stramenopiles had appeared by 1000 million years ago (Ma) [15], [16], [17], [18], [19], [20], [21]. Other putative early representatives of the heterokont algae, which provide further support for an early evolution of the group, are *Jacutianema* (ca. 750 Ma; [16]), *Germinosphaera* (750–700 Ma; [22]) and *Miaohephyton bifurcatum* (600–550 Ma; [23]). The first two fossils, which are considered form-taxa of *Palaeovaucheria* [16], are thought to be members of the class Xanthophyceae whereas *Miaohephyton bifurcatum* is considered to belong to Phaeophyceae (i.e. brown algae). Moreover, scales, similar to those seen in modern chrysophytes and structures resembling centric diatom valves, have been recovered from 811.5–717.4 Ma deposits in northwestern Canada [18], [24], [25], and ‘modern-looking’ diatoms have been reported from Proterozoic and Early Paleozoic deposits [26], [27] (although we note that the claim for Proterozoic diatoms is not considered reliable by mainstream paleontologists; [28]). The view regarding the timeframe within which the pigmented heterokonts evolved varies substantially depending on whether one considers fossil xanthophytes, phaeophytes, chrysophytes, and bacillariophytes from the Proterozoic (possibly ranging from the late Mesoproterozoic through the Neoproterozoic) to be taxonomically resolved or not. One hypothesis is that the photosynthetic stramenopiles originated sometime in the Paleozoic [5] and diversified throughout the Mesozoic [29], [30] whereas a radically different position holds that the pigmented heterokont groups evolved in the Proterozoic [16], [18], [20], [23].

In light of this uncertainty, the goals of this investigation were to infer, using nuclear-encoded SSU rRNA sequences, the timeframe within which the major lineages of heterokont algae originated and diversified, and to assess the validity of putative Proterozoic xanthophyte/phaeophyte fossils in the context of the reconstructed time-calibrated phylogeny. To robustly determine the placement of fossil constraints for subsequent divergence time estimation, an initial phylogeny was constructed using a Bayesian model which accommodated both pattern (substitution model) heterogeneity and heterotachy [31], [32]. Dating analyses, taking into account uncertainty in topological structure, employed an uncorrelated relaxed clock model of lineage-specific rate-heterogeneity [33], and considered two general approaches of translating fossil ages into temporal constraints.

## Materials and Methods

### Taxon sampling and nucleotide alignment

The nuclear-encoded SSU rRNA was chosen as the molecular marker for inferring phylogenetic relationships among the major ochrophyte lineages and the timeframes within which the photosynthetic heterokonts originated and diversified. Utilization of this gene allowed for the most expansive taxonomic sampling of the autotrophic stramenopile classes, including the non-photosynthetic oomycetes which are thought to be the closest living relatives of the ochrophytes [3], [8], [10], [11], [14]. Incorporating the immediate sister-taxon is imperative for gaining increased accuracy in elucidating the time period within which a given lineage evolved (i.e. it allows for the estimation of both stem- and crown-ages). In addition to the non-photosynthetic stramenopiles, we used representatives of the dinoflagellates, haptophytes, ‘green plants’, and rhodophytes as outgroups and for calibration purposes. All the 135 nuclear-encoded SSU rRNA sequences used in the study were obtained from GenBank (for accession numbers, see Table S1 in supplementary information).

The software package DAMBE v4.5.55 [34] was utilized to manage the nucleotide data. The alignment of the nucleotide sequences was carried out using MAFFT v6 [35]. The default settings of the parameters were used (scoring matrix value: 200PAM/K = 2; gap opening penalty = 1.53; offset value = 0.00). The alignment strategy implemented was L-INS-i [36]. The alignment is available from the corresponding author upon request.

### Phylogenetic tree reconstruction

In an attempt to reduce bias in phylogenetic inference, we employed a joint model that accommodates both rate- (heterotachy; [32]) and pattern-heterogeneity [31] as implemented in the program BayesPhylogenies (available from http://www.evolution.rdg.ac.uk/BayesPhy.html). A reversible-jump Markov chain Monte Carlo (rjMCMC) algorithm was used to determine how many distinct rate-variation patterns among sites and branch length parameters (with a maximum of two parameters for each branch) were required to optimally describe the empirical data matrix. In addition to potentially indentifying regions of the tree where phylogenetic reconstruction might be misled (for example, due to a high degree of heterotachy), an initial well-resolved tree was required to guide the placement of fossil calibrations in the divergence time analyses (below). A General Time Reversible (GTR) model of nucleotide substitution with discretized gamma-distributed rate variability (with 4 rate categories; γ_4_) was employed throughout. This is slightly simpler than the model implemented in divergence time estimation (GTR + γ_4_ + I; below), as the authors of BayesPhylogenies recommended against estimating the proportion of invariant sites. Five independent MCMC analyses (each with 1 chain running for 10^6^ generations, sampling every 10^3^ generations) were conducted to approximate the posterior distribution of phylogenetic trees, and post-burnin samples (with burnin set to 10%) from all analyses were combined for parameter summary. Convergence of the MCMC runs was assessed graphically by examining the cumulative posterior and between-run variation in split frequencies [37] using the on-line tool AWTY [38].

### Joint estimation of divergence times and phylogenetic relationships

Divergence time estimation accommodating topological uncertainty was performed using the relaxed clock model of Drummond et al. [33] under GTR + Γ_4_ + I as implemented in the program BEAST v1.5.3 [39]. Unlike most other relaxed clock methods available [e.g. 40], [41], this approach does not assume that rates are necessarily autocorrelated across the tree in an ancestor-descendant fashion; rather, branch-specific relative rates are drawn from a lognormal distribution, the mean and standard deviation of which are estimated from the data via MCMC sampling. A birth-death diversification process was used as a prior on the distribution of node heights. Tree topology and divergence times were estimated simultaneously, although for some internal nodes monophyly was enforced to facilitate the placement of prior age calibration distributions (see below). Six replicate runs of 10^7^ generations were performed for each analysis, sampling every 5×10^4^ generations. Convergence, mixing, and effective sample sizes (ESS) were monitored through the use of Tracer v1.5 [42]. Post-burnin samples were combined across runs to summarize parameter estimates.

### Temporal calibration constraints

**Figure 1:**
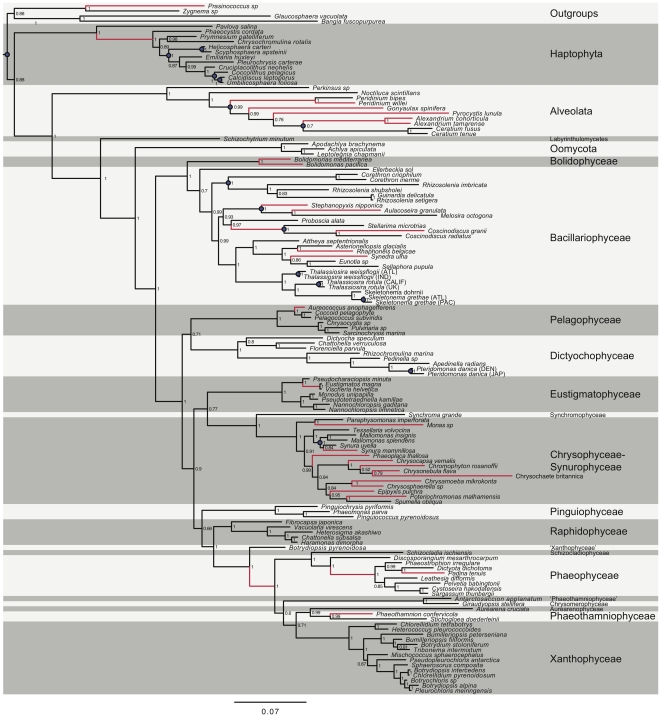
Consensus tree inferred from the Bayesian joint rate- and pattern-heterogeneity model. Consensus tree inferred from the Bayesian joint rate- and pattern-heterogeneity model [31], [32]. Numbers next to each node indicate inferred posterior clade probabilities. Red branches indicate those lineages inferred to having a greater than a 50% probability of having two distinct lengths in the posterior sample. The scale bar shows the expected number of substitutions per site. Blue circles indicate nodes with explicit temporal constraints (see Table 1).

Prior age calibration distributions are given in Table 1, and the positions of these constraints in the tree are indicated in Fig. 1. The node uniting the C_25_ HBI alkene producing rhizosolenids and the *Corethron* lineage was treated as a bounded constraint (91.5+/−1.5 Ma) because the sudden rise of the C_25_ HBI alkene in the geological record has been dated to have occurred between 90 and 93 Ma [43]. This time distribution was mimicked in the BEAST analyses through specifying a normal temporal prior. According to Haug and Tiedemann [44], the final closure of the Isthmus of Panama occurred sometime between 3.6 and 2.7 Ma. Nodes relevant to this geological event were assigned a normal temporal prior reflecting this range. The root node age was modeled with a uniform prior ranging between 1630 and 1160 Ma. This timeframe, which is also supported by the fossil occurrence of *Bangiomorpha* [45], is based on the estimated divergence between the red and green algal lineages obtained in a molecular phylogenetic dating analysis carried out by Hackett et al. [46].

**Table 1:** Temporal calibration constraints used in the divergence time analyses.

Higher taxon	Constrained node^a^	Age (Ma)^b^	Prior^c^	References
Bacillariophyceae	*Thalassiosira rotula* (UK) vs. (CALIF)	3.6–2.7^d^	N(3.15, 0.3)	[44]
	*Thalassiosira weissflogii* (ATL) vs. (IND)	3.6–2.7^d^	N(3.15, 0.3)	[44]
	*Skeletonema grethae* (PAC) vs. (ATL)	3.6–2.7^d^	N(3.15, 0.3)	[44]
	*Rhizosolenia setigera*, *Corethron inerme*	93–90^e^	N(91.5, 0.8)	[43]
	*Coscinodiscus granii*, *Stellarima microtrias*	Ca. 100^f^	1) E(6.0, 100); 2) LN(2.5, 0.5, 100)	[97], [98]
	*Melosira octogna*, *Stephanopyxis nipponica*	Ca. 100^g^	1) E(4.0, 100); 2) LN(2.0, 0.75, 100)	[98]
Dictyochophyceae	*Pteridomonas danica* (DEN) vs. (JAP)	3.6–2.7	N(3.15, 0.3)	[44]
Synurophyceae	*Synura uvella*, *Mallomonas insignis*	Ca. 49–40^h^	1) E(1.0, 49); 2) LN(2.0, 0.5, 49)	[99]
Haptophyta	*Umbilicosphaera folios*, *Calcidiscus leptoporus*	Ca. 24–16	1) E(1.0, 24.0); 2) LN(2.0, 0.5, 24)	[100], [101]
	*Helicosphaera carteri, Scyphosphaera apesteinii*	Ca. 32	1) E(1.0, 32); 2) LN(2.0. 0.5, 32)	[100]
	*Coccolithus pelagicus*, *Calcidiscus leptoporus*	Ca. 65^i^	1) E(1.0, 65); 2) LN(2.0, 0.5, 65)	[100], [102]
	*Helicosphaera carteri, Calcidiscus leptoporus*	Ca. 220–204^j^	1) E(4.0, 220); 2) LN(2.0, 0.75, 220)	[103]
Dinophyceae	*Ceratium fusus*, *Alexandrium tamarens*	Ca. 145^k^	1) E(4.0, 145); 2) LN(2.0, 0.75, 145)	[104]
	Peridiniales, *Alexandrium tamarens*	Ca.190^k^	1) E(4.0, 190); 2) LN(2.0, 0.75, 190)	[104]
Root	(Rhodophyta, Viridaeplantae), Ingroup	1630–1160^l^	U(1630–1160)	[45], [46]

a Calibrated node corresponds to the most recent common ancestral node of the listed taxa (see Fig. 1 for position of calibrated nodes). UK  =  United Kingdom; CALIF  =  California; ATL  =  Atlantic Ocean; IND  =  Indonesia; PAC  =  Pacific Ocean; DEN  =  Denmark; JAP  =  Japan.

b Fossil or molecular estimates taken from the literature. Ma  =  millions of years ago.

c Priors used for temporal constraints. N  =  Normal(mean, standard deviation); E  =  Exponential(mean, offset); LN  =  Lognormal(mean, standard deviation, offset). Two analyses were performed: 1) the majority of temporal constraint priors were exponentially-distributed, and 2) those same nodes were instead described by lognormal priors; the remaining constraint priors (U and N) were not altered across analyses. See text for explanation.

d Closure of the Panamanian Isthmus.

e *Rhizosolenia*-*Corethron* divergence are based on the estimated time of the abrupt increase in the C_25_ HBI alkene.

f First appearance of the genus *Coscinodiscus* in the fossil record.

g First appearance of the genus *Melosira* in the fossil record.

h First appearance of *Synura uvella* and *Mallomonas insignis* in the fossil record.

I First appearance of the genus *Coccolithus* in the fossil record.

j The origin of haptophyte calcification.

k Dates from fossil events in linearized tree [104].

l Inferred molecular divergence between (Rhodophyta, Viridaeplantae) and the ingroup [46]. Also supported by the fossil *Bangiomorpha* [45].

The constraints above were used in all divergence time analyses. For nodes of less precise ages (e.g. those that are known from one or few exceptional fossils) we opted to assess the sensitivity of inferred results through considering two distinct calibration procedures (Table 1). In the first set of analyses, narrow exponential distributions defined prior temporal constraints, the minimum (or ‘offset’) of which corresponded to the age of the relevant fossil. This approach lends high credence to a literal reading of the fossil record, as the highest prior probability is placed on the age of the fossil itself, while the rest of the prior mass lies adjacent to that age. In the second set of analyses, these same calibrated nodes were instead modelled with broader lognormal distributions, admitting an expected lag between cladogenesis and diagnosable fossil deposition (see Fig. 1 of [47]). For both sets of analyses, older constraints were considered less certain and hence were modelled with broader distributions (Table 1). Analyses implementing these alternative calibration sets allowed for the inspection of the influence of calibration choice on resulting divergence time estimates; in essence, these priors were constructed to mimic those that might be used by two investigators who disagree on the temporal precision of divergence time estimates derived from the fossil record. In addition, analyses were conducted for both temporal prior scenarios without data (2 replicates of 5×10^8^ generations) for the purpose of exploring joint-prior space (i.e. the joint distribution of all asserted temporal constraints in concert with the birth-death prior on non-constrained nodes). A comparison of posterior and joint-prior estimates gives an indication of the information content in the data; if these estimates are identical then the empirical data contain no information regarding the relevant parameters (in other words, the posterior is simply ‘recovering the prior’).

## Results and Discussion

### Phylogenetic relationships among the major pigmented heterokont lineages

**Figure 2:**
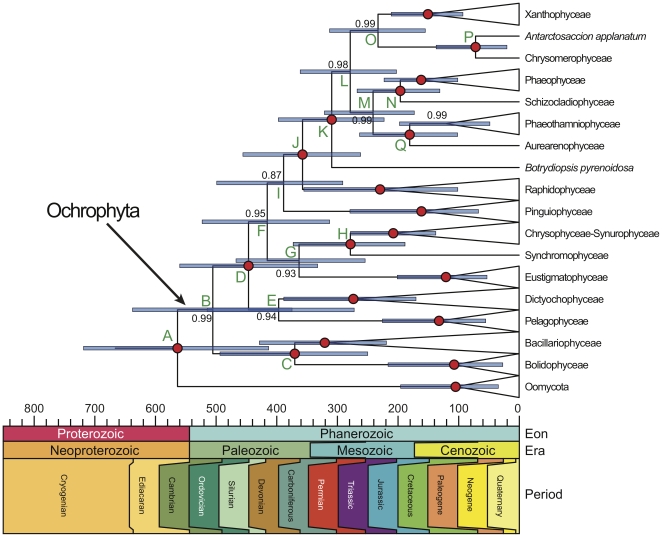
Maximum clade credibility chronogram. Maximum clade credibility chronogram derived from the summary of post-burnin samples from six independent BEAST analyses utilizing an uncorrelated lognormal relaxed clock model and lognormal temporal constraint priors (see text for explanation). Nodes are plotted as mean divergence time estimates (Ma), and blue horizontal bars represent 95% posterior credible intervals. Numbers in the tree diagram indicate posterior clade probabilities, with red circles representing nodes with posterior probability equal to 1.0. Green letters indentify major nodes whose age estimates across analyses are provided in Table 2. For legibility, major classes are collapsed and subsequent outgroups are not shown.

In trees derived from the BayesPhylogenies and BEAST analyses, the pigmented heterokonts formed a monophyletic group and a sister-lineage relationship with the oomycetes with high support values (Figs. 1, 2, S1, S2; see also [14]).

The consensus phylogeny derived from analyses employing the joint rate- and pattern-heterogeneity model [31], [32] in BayesPhylogenies yielded generally high posterior probabilities for the nodes uniting the major ochrophyte groups (Fig. 1). All nodes used for calibrating the divergence time analyses (Table 1) received posterior probabilities values of 1.0, except the one uniting the *Ceratium* and *Alexandrium* lineages (Dinophyceae) which had a posterior probability of 0.7 (Fig. 1). The fact that the great majority of the calibration nodes received posterior probability values of 1.0 justified fixing their phylogenetic relationships in the divergence time analyses. Branches exhibiting evidence for heterotachy (identified by having a greater than 0.5 posterior probability of having two distinct branch lengths) were non-randomly distributed, being mainly concentrated in lineages within Chrysophyceae-Synurophyceae, Bacillariophyceae/Bolidophyceae, and Alveolata (Fig. 1, red branches). However, the presence of heterotachy does not appear to have had any major influence on topological inference (see below).

The maximum-clade-credibility trees derived from the BEAST analyses using lognormal and exponential priors temporal priors (see *Methods* above) were identical in topology and almost indistinguishable in posterior clade probabilities (Figs. S1, S2). These near-identical inferences suggest that 1) both sets of analyses were sampling from the same distribution in tree-space, 2) both sets of analyses were run sufficiently long to produce a valid approximation to the stationary distribution (in addition to the evidence from large ESS values), and 3) alternative approaches to implementing temporal constraints had negligible influence on topological inference. However, these BEAST trees show one important topological discrepancy in regard to the topology obtained from BayesPhylogenies (Fig. 1). In the BEAST topology the Phaeothamniophyceae/Aurearenophyceae clade constitutes a sister-lineage to the clade uniting Schizocladiophyceae/Phaeophyceae (Fig. 2). In the consensus tree derived from the BayesPhylogenies analysis Phaeothamniophyceae/Aurearenophyceae instead shares a most recent common ancestor with Xanthophyceae (see also the combined data analysis in [48]). As the relevant branches characterizing differences across the trees are not inferred to involve heterotachy (Fig. 1), these dissonant results are likely explained through the accommodation of pattern heterogeneity [31] in the BayesPhylogenies analyses.

All trees reconstructed herein agree on inferred instances of paraphyly. Minor examples include genera in Bacillariophyceae (*Rhizosolenia* and *Thalassiosira*) and Xanthophyceae (*Chlorellidium*, *Bumilleriopsis*, and *Botrydiopsis*). However, more notable instances exist. For example, in all trees *Antarctosaccion applanatum* is removed from the remaining ‘Phaeothamniophyceae’ species (*Phaeothamnion confervicola* and *Stichogloea doederleinii*), instead forming a sister-relationship (with posterior probability 1.0) with Chrysomerophyceae representative *Giraudyopsis stellifera*, a result that has independently been inferred from 18S rDNA [48]. Additionally, our analyses reject the clustering of *Botrydiopsis pyrenoidosa* with Xanthophyceae [49], or indeed with any of the other classes, suggesting that additional major evolutionary lineages may exist among the photosynthetic stramenopiles. Finally, our results indicate that Chrysophyceae and Synurophyceae are paraphyletic taxa (for discussion, see [50]), due to the *Chrysosphaerella* lineage (Synurophyceae) forming a sister-relationship with *Chrysamoeba mikrokonta* (Chrysophyceae) with posterior probability 1.0 in all analyses.

Previous phylogenies of the major photosynthetic stramenopile lineages, derived mainly from nuclear-encoded SSU rRNA, *rbcL* sequences and combined data sets (both molecular and non-molecular data), are generally poorly resolved, and thus few consensus relationships exist across studies [2], [3], [11], [12], [13], [48], [51], [52], [53], [54]. A likely explanation for the observed discrepancies in phylogenetic relationships among the major groups of heterokont algae concerns the sensitivity of inferences to differences in both ingroup and outgroup sampling [13]. A recent multigene phylogenetic study [8] with relatively sparse taxon-sampling (*n* = 35) showed improved phylogenetic resolution among some of the pigmented heterokont classes relative to previous studies, suggesting that extended gene-sampling may offset difficulties arising from limited taxon-sampling. Nevertheless, the phylogenetic positions of several classes (Pinguiophyceae, Bacillariophyceae, Chrysophyceae, Raphidophyceae, and Eustigmatophyceae) remained poorly supported (posterior probabilities 0.64-0.69; [8; their Fig. 3]). The trees obtained in the present study, particularly those inferred from the BEAST analyses, reveal additional well supported branching orders among the ochrophyte classes. We attribute this higher support to a sophisticated modelling of the data, but also to relatively dense and targeted taxon-sampling design.

Both the multigene phylogeny published by Riisberg et al. [8] and the trees inferred here suggest that Bacillariophyceae, Pelagophyceae, and Dictyochophyceae are among the earliest ochrophyte classes to evolve and that some of the most recent radiations included groups such as Xanthophyceae, Phaeophyceae, and Phaeothamniophyceae. However, the exact branching orders among these major lineages differed between the two studies. In particular, the phylogenetic position of Raphidophyceae (inferred here with high posterior support; Figs. 1, 2) is incompatible, although support for the phylogenetic arrangement in the former study is poor [8]. Previous analyses based both on SSU rRNA, *rbcL* sequences and combined data, have been unable to identify a well corroborated relationship for this taxon [2], [8], [12], [13], [50], [51], [52], [53], [54]. Resolution of these and other conflicts will require more expansive taxon and gene sampling.

### When did the extant heterokont algae lineages evolve?

Replicate BEAST MCMC analyses converged to the same likelihood for both lognormal and exponential calibration sets (mean posterior model log likelihood = −48906; see *Methods* above) and showed high (>400) ESSs for all sampled parameters. The estimated coefficient of variation was nearly identical across exponential (mean  = 0.85, 95% credible interval (CI)  = 0.73–0.98) and lognormal (mean  = 0.86, 95% CI  = 0.74–0.98) calibration priors, despite the narrower temporal constraints employed in the former analyses. Both sets of analyses thus strongly suggest rejection of a strict molecular clock (i.e. the inferred rates vary by 85% of the mean over the tree). Likewise, the calculated covariance from exponential (mean  = 0.086, 95% CI  = −0.035–0.21) and lognormal (mean  = 0.082, 95% CI  = −0.035–0.204) age priors, both overlapping zero, agree that there is no strong evidence for an ancestor-descendant autocorrelation of rates in the phylogeny. These two findings justify our choice of BEAST for dating purposes over alternative autocorrelated approaches. A comparison of inferred posterior and prior (i.e. no data) divergence times (Table 2) indicates a substantial amount of historical signal in the present alignment, as revealed by posterior estimates being generally younger and more precise than corresponding prior estimates. We can thus be assured that our inferences are not based on a recovery of the prior probabilities, but rather from historical signal extracted from the empirical data.

**Table 2:** Estimated divergence times (Ma) among the major photosynthetic heterokont lineages.

		Exponential age priors			Lognormal age priors		
Node^a^	Cladogenetic event^b^	Prior age^c^	Posterior age^d^	P(clade)^e^	Prior age^c^	Posterior age^d^	P(clade)^e^
A	stem origin Ochrophyta	874 (1262,490)	543 (705,396)	1.0	885 (1272,511)	564 (719,414)	1.0
B	stem origin (Bolidophyceae+Bacillariophyceae)	767 (1124,428)	486 (619,359)	0.99	780 (1142,446)	506 (638,375)	0.99
C	Bolidophyceae vs. Bacillariophyceae	559 (905,256)	353 (473,238)	1.0	572 (913,263)	370 (494,250)	1.0
D	stem origin (Dictyochophyceae+Pelagophyceae)	668 (1000,364)	428 (543,322)	1.0	683 (1009,379)	447 (560,333)	1.0
E	Dictyochophyceae vs. Pelagophyceae	432 (757,131)	382 (506,270)	0.93	443 (764,132)	397 (515,272)	0.94
F	(Eust., Synch., Chrys.-Synur.) vs. more recent ochrophytes	583 (888,313)	398 (506,297)	0.94	597 (900,327)	416 (523,313)	0.95
G	stem origin Eustigmatophyceae	451 (724,195)	349 (459,250)	0.93	466 (745,212)	363 (467,254)	0.93
H	stem origin Synchromophyceae	342 (581,127)	268 (361,177)	1.0	356 (598,139)	279 (373,188)	1.0
I	stem origin Pinguiophyceae	488 (765,239)	371 (474,275)	0.87	502 (781,257)	389 (499,291)	0.87
J	stem origin Raphidophyceae	415 (658,189)	341 (439,248)	1.0	428 (677,205)	358 (456,262)	1.0
K	stem origin *Botrydiopsis*	353 (572,153)	294 (382,215)	1.0	365 (590,168)	309 (397,223)	1.0
L	(Xanthophyceae+RT) vs. (Phaeothamniophyceae+RT)	305 (502,130)	265 (346,194)	0.98	316 (514,137)	279 (361,202)	0.98
M	(Phaeoth., Aur.) vs. (Schiz., Phaeoph.)	232 (402,74)	229 (299,161)	0.99	242 (419,84)	241(321,172)	0.99
N	Schizocladiophyceae vs. Phaeophyceae	172 (321,46)	186 (253,124)	1.0	180 (332,50)	196 (267,131)	1.0
O	stem origin Xanthophyceae	246 (422,93)	222 (302,151)	0.99	257 (435,98)	233 (313,155)	0.99
P	Chrysomerophyceae vs. *Antarctosaccion applanatum*	76 (221,1)	68 (131,20)	1.0	80 (230,1)	72 (137,20)	1.0
Q	Phaeothamniophyceae vs. Aurearenophyceae	106 (247,2)	171 (251,99)	1.0	113 (261,2)	181 (263,102)	1.0

a Labels correspond to those presented in Fig. 2.

b RT  =  related taxa; Eust.  =  Eustigmatophyceae; Synch  =  Synchromophyceae; Chrys.-Synur.  =  Chrysophyceae-Synurophyceae; Phaeoth.  =  Phaeothamniophyceae; Aur.  =  Aurearenophyceae; Schiz.  =  Schizocladiophyceae; Phaeoph.  =  Phaeophyceae.

c Mean divergence times (95% credible intervals) derived from 2 replicates of 5×10^8^ generation analyses exploring only joint-prior space (i.e. no data). See text for explanation.

d Mean divergence times (95% credible intervals) derived from 6 replicates of 10^7^ generation analyses.

e Posterior clade probabilities over all post-burnin trees across replicate analyses.

A number of hypotheses have been proposed regarding the timing of evolution of the extant pigmented heterokonts. One view (the Paleozoic hypothesis) developed from analyses of nuclear 18S rRNA sequences holds that the photosynthetic stramenopiles originated between 498 and 293 Ma [5], [29] and subsequently diversified throughout the Mesozoic [29], [30]. Another position, supported by the fossil record of putative xanthophyte algae, claims that the group could have originated as far back in time as the late Mesoproterozoic/early Neoproterozoic [16], [17], [20], [21]. This more ancient timeframe has been corroborated from molecular clock analyses of *rbcL* data [29].

Our results provide evidence for an intermediate Neoproterozoic-Paleozoic timeframe, the divergence between the oomycetes and the pigmented heterokonts inferred to have occurred between the mid-Neoproterozoic and Early Devonian (lognormal priors: mean  = 571 Ma, 95% CI  = 735–434 Ma; exponential priors: mean  = 529 Ma, 95% CI  = 673–396; Fig. 2, node A; Table 2). Although this timescale is somewhat older than what is suggested by the Paleozoic hypothesis, posterior CIs nevertheless overlap with timescales estimated previously. However, these results are entirely irreconcilable with the alternative Meso-Neoproterozoic hypothesis. The timescale presented here reveals a discrepancy of several hundred million years between the oldest (∼1000 Ma) putative pigmented stramenopile fossil [16], [17], [20], [21] and the inferred origination time of the extant ochrophyte clade. Such a degree of dissonance might normally imply that an Ochrophyta+Oomycota stem group representative had mistakenly been interpreted as belonging to the crown group. However, the fact that these fossils have been assigned to much younger, relatively derived clades renders this hypothesis improbable, as it would require multiple secondary losses of characters across the ochrophyte tree. A more likely explanation would seem to be that these fossils have been misidentified as being crown group members due to the independent evolution of ‘derived’ characteristics in relatively distantly related taxa ([23]; see below). For example, convergent evolution in unicellular eukaryotes has been documented between the centric diatoms and the dinoflagellate genus *Prorocentrum* as well as between polycysteine radiozoans and the silicoflagellate genus *Dictyocha* [55].

### Inferred evolutionary timescale of the pigmented heterokont classes with a fossil record

The brown algae are one of the most ecologically diverse groups of primary producers, exhibiting a wide variety of forms ranging from simple filaments to large complex plant-like organisms. A number of ‘brown algae’ of uncertain taxonomic status have been discovered in Precambrian, Paleozoic and Mesozoic deposits [23], [56], [57], [58], [59], [60]. Our analyses indicate that the Phaeophyceae and Schizocladiophyceae lineages most likely diverged in the Lower Jurassic (lognormal priors: mean  = 196 Ma, 95% CI  = 268–131 Ma; exponential priors: mean  = 186 Ma, 95% CI  = 253–125; Fig. 2, node N; Table 2). Several previous estimates exist in the literature regarding this cladogenetic event. Analyses of numerous plastid protein-coding genes yielded comparatively younger estimates (124–62 Ma; [61]), while both 18S (155 Ma; [29]) and 5S (200 Ma; [62]) rRNA gene data inferred timescales roughly in agreement with the present study. Regardless, these estimates all indicate a clear conflict with regard to the presence of putative Neoproterozoic and early Paleozoic brown algae, with molecular estimates post-dating the geological age of the putative phaeophyte fossil *Miaohephyton bifurcatum* (600–550 Ma; [23]) by roughly 300 million years. It seems unlikely that this late Neoproterozoic fossil is a true member of the living Phaeophyceae since this group often appears as one of the most recent clades in stramenopile trees ([2], [3], [8]; this study). Given our temporal reconstruction (Fig. 2), reconciliation of *Miaohephyton* with existing molecular divergence time estimates does not appear possible without postulating widespread secondary losses of characters from all lineages except Phaeophyceae. Instead, a more probable scenario is that *Miaohephyton* has been misidentified as belonging to the phaeophyte crown group due to convergent evolution of brown algae-like characteristics [23] in green and red algae, or perhaps in an early photosynthetic stramenopile lineage which left no descendants.

In general Class Xanthophyceae is characterized by having a poor fossil record [56]. Our analyses suggest that this taxon most likely originated in the Middle Triassic (lognormal priors: mean  = 233 Ma, 95% CI  = 314–155 Ma; exponential priors: mean  = 223 Ma, 95% CI  = 301–151; Fig. 2, node O; Table 2). However, as mentioned above, putative xanthophyte fossils dating from ∼1000 Ma [16], [19], [20], [21] would suggest that the present investigation underestimated the time of the evolution of the extant Xanthophyceae by more than half a billion years. Similar to the molecular/fossil time discrepancy regarding Phaeophyceae above, since xanthophytes are inferred to be one of the more derived clades in heterokont phylogenies ([2], [3], [8]; this study) it is highly unlikely that the group is of the antiquity required by these fossils. Our results thus reject the interpretation of these fossils as vaucheriacean from both temporal and topological perspectives. Extant *Vaucheria*-like characteristics seen in *Paleovaucheria* and other late Mesoproterozoic/early Neoproterozoic xanthophyte fossils are therefore more likely the result of convergent evolution that occurred in an early extinct ochrophyte lineage, or possibly (due to the great antiquity of the fossils involved) in an old non-stramenopile group.

There is not much known about the fossil history of the chrysophytes, which include both Chrysophyceae and Synurophyceae [50]. Lower Cretaceous strata (Aptian-Albian, 125–99 Ma) are thought to contain the earliest fossil record of the chrysophytes [63], [64]. However, scales, similar to those seen in modern chrysophytes, have also been reported from 811.5–717.4 Ma deposits in northwestern Canada [18], [24], [25]. Our analyses indicate that chrysophytes most likely originated in the Permian (lognormal priors: mean  = 279 Ma, 95% CI  = 373–189 Ma; exponential priors: mean  = 268 Ma, 95% CI  = 363–179; Fig. 2, node H; Table 2). Thus, we infer from our molecular data that the extant chrysophytes evolved more than 50 million years earlier than is suggested by reliable fossil evidence from the Cretaceous. However, if one considers the putative Precambrian chrysophyte scales, the time of origin of the Chrysophyceae/Synurophyceae clade was underestimated in this study by at least 220 million years.

Class Dictyochophyceae, which once was considered to be a member of the class Chrysophyceae, includes the silicoflagellates (order Dictyochales), a group characterized by formation of a silicified skeleton. Due to the silicified structures these organisms possess a fossil record starting in the Early Cretaceous (145.5–99.6 Ma; [65]). The age estimates obtained herein suggested that Dictyochophyceae evolved much earlier than the paleontological record indicates, the divergence from its sister-lineage (Pelagophyceae) taking place between the Early Cambrian and Permian (lognormal priors: mean  = 397 Ma, 95% CI  = 520–279 Ma; exponential priors: mean  = 382 Ma, 95% CI  = 497–264; Fig. 2, node E; Table 2).

The pigmented stramenopile taxon with the most extensive fossil record is the Class Bacillariophyceae (also recognized as the Division Bacillariophyta). Diatoms are known from sediments as old as the earliest Cretaceous (approx. 140 Ma; [66]) and they may go as far back as the Early Jurassic (approx. 190 Ma; [67], [68]). Previous studies assuming a global molecular clock have suggested that the Bacillariophyceae clade evolved in the Early Cretaceous (approx. 135 Ma) and as early as in the Middle Triassic (approx 240 Ma; [29], [69], [70]). In a molecular phylogenetic dating analysis which did not assume rate constancy, Sorhannus [71] found that the diatoms may have diverged from their sister-lineage between the late Permian (267 Ma) and the Middle Jurassic (162 Ma). However, it has subsequently been demonstrated that the dating method used in Sorhannus (PATHd8 [72], [73]) is statistically biased, generating overly young and precise divergence time estimates [74], and has produced statistically significant dissonant inferences for empirical data when compared to more vetted analytical methods [47], [75]. In the present investigation these boundaries have been pushed considerably further back in time (lognormal priors: mean  = 370 Ma, 95% CI  = 493–251 Ma; exponential priors: mean  = 354 Ma, 95% CI  = 474–238; Fig. 2, node C; Table 2). These new results suggest that the diatom lineage evolved sometime near the Devonian-Carboniferous transition, and that the fossils of many diatom groups (e.g. ‘pennate’ diatoms) could be much older than the currently known paleontological record has indicated [66], [71]. Despite our results indicating that representatives of Bacillariophyceae may already have existed in the early Paleozoic, modern looking diatoms reported by Sieminska and Kwiecinska [26], [27] from this time period are likely to be ‘contaminants’ from younger strata because the earliest known diatoms are morphologically rather different from modern diatoms. Moreover, many workers are unconvinced that many of the fossils reported from the early Paleozoic are actually diatoms [28].

### Inferred evolutionary timescale of the pigmented heterokont classes without a fossil record

Most ochrophyte classes generally lack a fossil record, and tend to be currently composed of relatively few species. Major pigmented heterokont lineages that are absent from the paleontological record include Aurearenophyceae, Schizocladiophyceae, Synchromophyceae, Bolidophyceae, Phaeothamniophyceae, Chrysomerophyceae, Pelagophyceae, Eustigmatophyceae, Pinguiophyceae, and Raphidophyceae. This is reflected in the distribution of temporally-constrained nodes in the present study (Fig. 1). Andersen [2] posed the question of whether these groups are ancient and consist of a few remnant species, or if they are newly evolved groups that have not yet radiated. The present study indicates that these lineages originated at considerably different periods (Fig. 2; Table 2), suggesting that neither of these possibilities likely holds generally across all clades [76], [77].

The earliest divergence event within the photosynthetic stramenopiles occurred between Bacillariophyceae/Bolidophyceae and a ‘super-clade’ consisting of the remaining extant pigmented heterokont lineages near the Cambrian-Ordovician transition (lognormal priors: mean  = 506 Ma, 95% CI  = 636–373 Ma; exponential priors: mean  = 486 Ma, 95% CI  = 619–362; Fig. 2, node B; Table 2). Eustigmatophyceae diverged from a lineage consisting of Synchromophyceae/Chrysophyceae –Synurophyceae between the Middle Ordovician and the late Permian (Fig. 2, node G; Table 2). Other major lineages originating during this time period (467–255 Ma) include Raphidophyceae, Pinguiophyceae and the lineage represented by *Botrydiopsis pyrenoidosa*. Schizocladiophyceae, Phaeophyceae, Phaeothamniophyceae, and Aurearenophyceae are inferred to have most probably originated in the Triassic and Jurassic Periods (Fig. 2; Table 2). In contrast, the only divergence inferred to have taken place entirely in the Phanerozoic was that between *Giraudyopsis stellifera* (Chrysomerophyceae) and *Antarctosaccion applanatum* (lognormal priors: mean  = 72 Ma, 95% CI  = 137–20 Ma; exponential priors: mean  = 68 Ma, 95% CI  = 131–20; Fig. 2, node P; Table 2).

### Robustness of molecular divergence time estimates

All molecular dating approaches make assumptions [78], [79], [80], [81], and these should be considered critically on a per data set basis. For example, most currently available approaches assume that the phylogeny is known without error. However, if this assumption is considered untenable (for example with poor nonparametric bootstrap or posterior probability values) then any inferences made under this assumption should be regarded with skepticism. In the present study the great majority of nodes defining relationships among the heterokont algal classes are well supported (Fig. 1). This can be considered an advance in the attempt to infer evolutionary relationships between the major photosynthetic stramenopile taxa since many studies, using various genes and combined data sets (both molecular and non-molecular data), have often shown poorly resolved phylogenetic positions of the classes [2], [12], [13], [51], [52], [53], [54]. Nevertheless, we opted to relax the fixed-topology assumption in order to investigate the degree of topological congruency across analyses/models. Indeed, our BayesPhylogenies and BEAST trees disagree importantly in the placement of the Phaeothamniophyceae/Aurearenophyceae clade (Figs. 1, 2). Although the accommodation of topological uncertainty comes at the expense of less precise inferences (through considering a broader portion of parameter space), we regard this as a more honest approach to presenting the historical signal possessed in the empirical data.

A second important issue concerns the treatment of temporal constraints. Dating analyses often implement calibration dates that assume a close correspondence between the first appearance of morphospecies in the fossil record and genetic speciation [29], [69], at the extreme assigning to a node to the age of the relevant fossil. However, when morphological differentiation and genetic speciation are decoupled cladogenesis can potentially take place appreciably earlier than detectable species level morphological delineation (see Fig. 1 in [47]). Such a situation is expected to result in a bias towards younger paleontological divergence time estimates [82]. Operationally, this is a concern since recent molecular evolutionary studies have demonstrated that unicellular eukaryotes can exist as cryptic/semi-cryptic species, such as the diatoms, *Thalassiosira weissflogii* [83], *Ditylum brightwellii* [84], *Cyclotella meneghiniana* [85], *Pseudo-nitzschia delicatissima*/*pseudodelicatissima* [86], and the foraminiferan *Orbulina universa* [87]. These organisms appear to have differentiated considerably at the molecular genetic level without any major discernable morphological differences.

In the present study we considered alternative (tight) exponential and (broad) lognormal calibration priors to investigate the sensitivity of resulting inferences to choice of temporal prior distributions. Not surprisingly, exponential priors (lending more credence to a strict reading of the fossil record) generated generally younger and more precise inferred divergence times than the considerably broader lognormal priors (which model an expected lag between speciation and diagnosable fossil deposition). Nevertheless, the two sets of estimates are broadly congruent with considerably overlapping 95% CIs, suggesting for the present data set that (reasonable) alternative prior constructs are unlikely to significantly support dissonant temporal evolutionary hypotheses. Some of the calibration dates used here are not based directly on recognizing the first appearance of morphological species in the fossil record but rather on the time of the final closure of the Isthmus of Panama [44] and the correlation between the very abrupt increase of the C_25_ HBI alkene in the Turonian [43] and the rapid evolution of rhizosolenid diatoms (*Rhizosolenia* and *Guinardia* species). These are considered to be reliably dated geological events and provide important historical information in lineages with poor or absent fossil records. While we recognize that these alkenes (analogous to morphological characters, above) may have evolved following cladogenesis (rendering our divergence time estimates as overly young), because the nodes involved are all located near the tips of the tree, it appears quite unlikely that a speciation/alkene decoupling at this tree depth would seriously mislead our broader inferences regarding the earliest putative fossils from this group (see above).

A third issue in molecular dating involves the general modelling of among-lineage rate heterogeneity. For example, typical empirical molecular genetic alignments of non-trivial size are rarely fit by ‘global’ molecular clocks. Relaxed clock approaches offer a break from the unwarranted assumption of a global clock through allowing individual branches within a tree to have unique rates of molecular evolution. However, many of the available relaxed clock models [e.g. 40], [41] assume an autocorrelation of ancestor-descendent rates. Recent studies of virus, marsupial [33], mammal [88], fish [89], plant [90], [91], and avian [47] data sets indicate that empirical sequences tend to exhibit non-autocorrelated rates. Likewise, the posterior distribution of the coefficient of variation estimated here strongly renders a global molecular clock assumption untenable, and the calculated covariance among inferred branch rates suggest there is little evidence for an ancestor-descendant autocorrelation of rates in the phylogeny used in this study. Indeed, a rejection of autocorrelated rate-evolution is generally expected at deep taxonomic levels due to stochastic variation alone [33], [92].

Finally, a general issue for all phylogenetic studies concerns taxon and molecular sampling. In the present study we elected to maximize taxonomic sampling, as this has been demonstrated to be important in divergence time estimation [93], [94]. We recognize the limitations inherent in using a single locus for both phylogeny reconstruction (gene trees can differ from underlying species trees; [95]) and molecular dating (the pattern of rate-variation in a single locus may not be representative of the genome as a whole). In particular, our results reveal a general phenomenon in molecular dating where older nodes are less precisely estimated [96]. These older estimates in particular will benefit through the future addition of multiple unlinked loci. Nevertheless, we regard our results as an important step towards a robust temporal perspective on the origination and diversification of the autotrophic stramenopiles, and consider the validity of putative Proterozoic xanthophyte/phaeophyte fossils (differing by as much as half billion years or more from the timescale inferred here) as being strongly rejected by the data in hand.

### Summary and conclusions

The results of the divergence time analyses presented herein indicate that the first pigmented stramenopiles most likely evolved between the mid-Neoproterozoic and the Early Devonian. These estimates suggest that the radiation of the majority of the known heterokont algal classes occurred throughout the Paleozoic and in the Triassic/Jurassic of the Mesozoic. Our uncorrelated relaxed clock investigation gave rise to generally older origination times for most stramenopile classes relative to most previous fossil- and molecular genetic-based estimates. Nevertheless, these results are topologically and temporally incompatible with putative Mesoproterozoic/Neoproterozoic xanthophyte (*Palaeovaucheria*, *Jacutianema*, *Germinosphaera*) and phaeophyte (*Miaohephyton bifurcatum*) fossils, casting doubt on the taxonomic validity of these fossils. Elucidation of their taxonomic affinity is difficult because *Vaucheria*-like characteristics have most likely evolved in these extinct Proterozoic lineages independently of those seen in the extant forms of the genus *Vaucheria* (Xanthophyceae). Nevertheless, the degree of dissonance suggested by our results is such that if these fossils truly represent crown stramenopile lineages then we would have to conclude that ochrophyte molecular evolution proceeds in a fashion that is fundamentally incompatible with the uncorrelated relaxed clock model employed here. It is the implausibility of this scenario that we regard convergent morphological evolution as a more likely explanation.

## Supporting Information

Figure S1 Maximum clade credibility chronogram. Maximum clade credibility chronogram from BEAST analyses utilizing an uncorrelated lognormal relaxed clock model and lognormally-distributed temporal constraint priors (see main text for explanation). All included taxa are shown. Nodes are plotted as mean divergence time estimates (Ma), and blue horizontal bars represent 95% posterior credible intervals. Numbers in the tree diagram indicate posterior clade probabilities. Estimates are derived from the summary of post-burnin samples from six independent MCMC analyses.
(3.21 MB TIF)

Figure S2 Maximum clade credibility chronogram. Maximum clade credibility chronogram from BEAST analyses utilizing an uncorrelated lognormal relaxed clock model and exponentially-distributed temporal constraint priors (see main text for explanation). All included taxa are shown. Nodes are plotted as mean divergence time estimates (Ma), and blue horizontal bars represent 95% posterior credible intervals. Numbers in the tree diagram indicate posterior clade probabilities. Estimates are derived from the summary of post-burnin samples from six independent MCMC analyses.
(3.14 MB TIF)

Table S1 GenBank accession numbers of the 135 species used in the study
(0.12 MB DOC)
