# Supplementary figures and images for: A Molecular Genetic Timescale for the Diversification of Autotrophic Stramenopiles (Ochrophyta): Substantive Underestimation of Putative Fossil Ages
Source: PLoS One. 2010 Sep 16;5(9):e12759. doi: 10.1371/journal.pone.0012759 (PMC2940848; doi:10.1371/journal.pone.0012759)

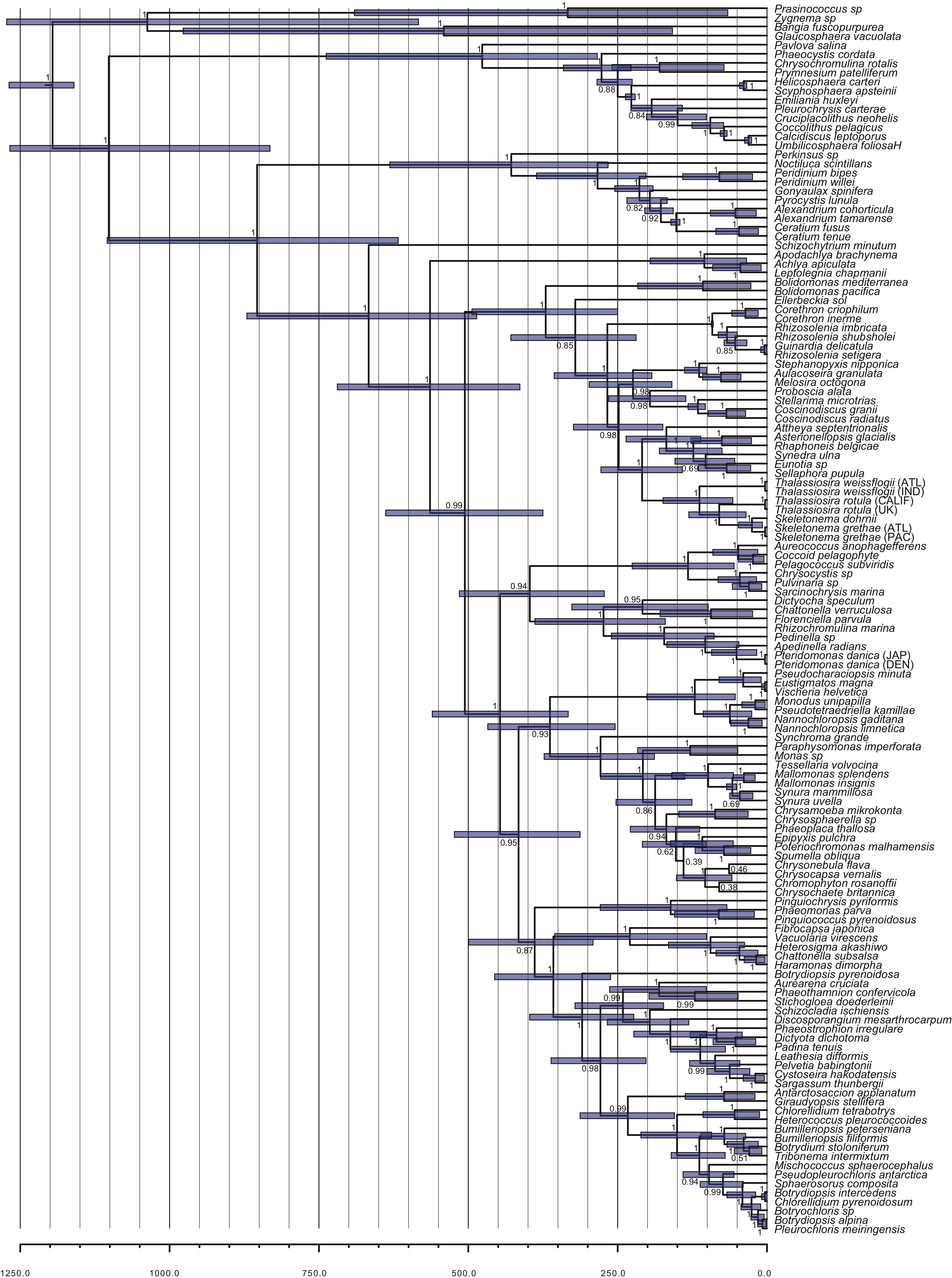

Supplement: Figure S1 — Maximum clade credibility chronogram. Maximum clade credibility chronogram from BEAST analyses utilizing an uncorrelated lognormal relaxed clock model and lognormally-distributed temporal constraint priors (see main text for explanation). All included taxa are shown. Nodes are plotted as mean divergence time estimates (Ma), and blue horizontal bars represent 95% posterior credible intervals. Numbers in the tree diagram indicate posterior clade probabilities. Estimates are derived from the summary of post-burnin samples from six independent MCMC analyses. (3.21 MB TIF) [file pone.0012759.s001.tif]

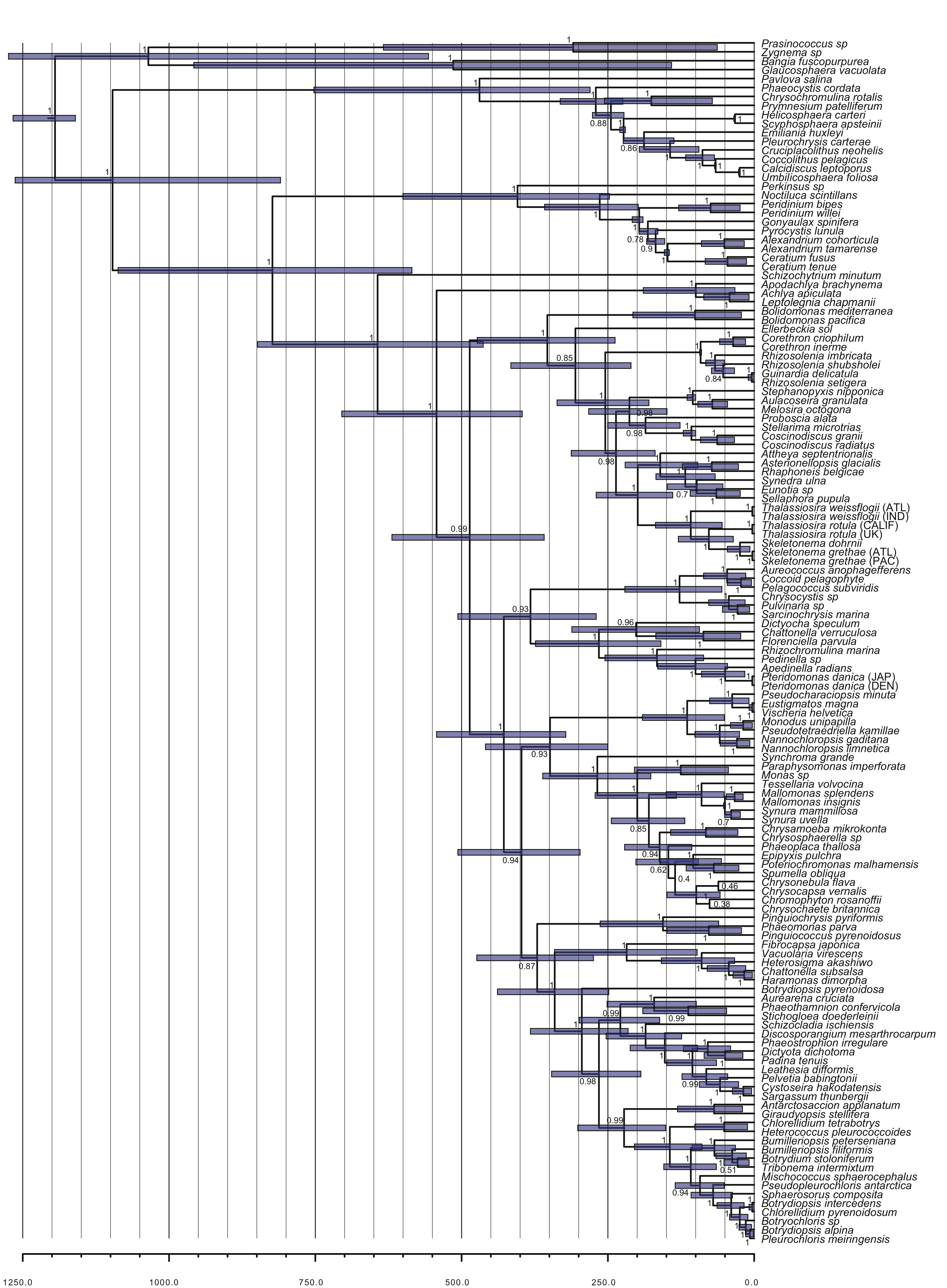

Supplement: Figure S2 — Maximum clade credibility chronogram. Maximum clade credibility chronogram from BEAST analyses utilizing an uncorrelated lognormal relaxed clock model and exponentially-distributed temporal constraint priors (see main text for explanation). All included taxa are shown. Nodes are plotted as mean divergence time estimates (Ma), and blue horizontal bars represent 95% posterior credible intervals. Numbers in the tree diagram indicate posterior clade probabilities. Estimates are derived from the summary of post-burnin samples from six independent MCMC analyses. (3.14 MB TIF) [file pone.0012759.s002.tif]
