# Supplementary material for: A Molecular Genetic Timescale for the Diversification of Autotrophic Stramenopiles (Ochrophyta): Substantive Underestimation of Putative Fossil Ages
Source: PLoS One. 2010 Sep 16;5(9):e12759. doi: 10.1371/journal.pone.0012759 (PMC2940848; doi:10.1371/journal.pone.0012759)
Supplement: Table S1 — GenBank accession numbers of the 135 species used in the study (0.12 MB DOC) [file pone.0012759.s003.doc]

| **Higher Taxon** | **Species** | **Accession Number** |
| --- | --- | --- |
| Aurearenophyceae | *Aurearena cruciata* | AB365192 |
| Bacillariophyceae | *Ellerbeckia sol* | AJ535174 |
| Bacillariophyceae | *Rhizosolenia setigera* | AY485508 |
| Bacillariophyceae | *Rhizosolenia shrubshrolei* | AY485510 |
| Bacillariophyceae | *Rhizosolenia imbricata* | AJ535178 |
| Bacillariophyceae | *Guinardia* *delicatula* | AJ535192 |
| Bacillariophyceae | *Proboscia alata* | AJ535181 |
| Bacillariophyceae | *Corethron criophilum* | X85400 |
| Bacillariophyceae | *Thalassiosira rotula* (CALIF) | AF462059 |
| Bacillariophyceae | *Thalassiosira rotula* (UK) | X85397 |
| Bacillariophyceae | *Thalassiosira weissflogii* (ATL) | AF374477 |
| Bacillariophyceae | *Thalassiosira weissflogii* (IND) | EF585582 |
| Bacillariophyceae | *Corethron inerme* | AJ535180 |
| Bacillariophyceae | *Stephanopyxis nipponica* | M87330 |
| Bacillariophyceae | *Coscinodiscus radiatus* | X77705 |
| Bacillariophyceae | *Asterionellopsis glacialis* | X77701 |
| Bacillariophyceae | *Synedra ulna* | AJ535139 |
| Bacillariophyceae | *Rhaphoneis belgicae* | X77703 |
| Bacillariophyceae | *Eunotia sp* | AJ535145 |
| Bacillariophyceae | *Sellaphora pupula* | AJ535155 |
| Bacillariophyceae | *Coscinodiscus granii* | AY485495 |
| Bacillariophyceae | *Stellarima microtrias* | AY485477 |
| Bacillariophyceae | *Melosira octogona* | AY485518 |
| Bacillariophyceae | *Skeletonema dohrnii* | AJ632210 |
| Bacillariophyceae | *Attheya septentrionalis* | AY485517 |
| Bacillariophyceae | *Aulacoseira granulata* | AY569584 |
| Bacillariophyceae | *Skeletonema* grethae (PAC) | AY684946 |
| Bacillariophyceae | *Skeletonema* grethae (ATL) | AJ632205 |
| Bolidophyceae | *Bolidomonas mediterranea* | AF123596 |
| Bolidophyceae | *Bolidomonas pacifica* | AF123595 |
| Phaeothamniophyceae | *Phaeothamnion confervicola* | AF044846 |
| Phaeothamniophyceae | *Stichogloea doederleinii* | AB365201 |
| Chrysomerophyceae | *Giraudyopsis stellifera* | U78034 |
| Schizocladiophyceae | *Schizocladia ischiensis* | AB085614 |
| Phaeophyceae | *Leathesia difformis* | AY232603 |
| Phaeophyceae | *Cystoseira hakodatensis* | AB011425 |
| Phaeophyceae | *Sargassum thunbergii* | DQ666483 |
| Phaeophyceae | *Pelvetia babingtonii* | AB011424 |
| Phaeophyceae | *Phaeostrophion irregulare* | AB117949 |
| Phaeophyceae | *Padina tenuis* | AF350239 |
| Phaeophyceae | *Dictyota dichotoma* | AB087107 |
| Phaeophyceae | *Discosporangium mesarthrocarpum* | AB252657 |
| Xanthophyceae | *Chlorellidium pyrenoidosum* | AJ579338 |
| Xanthophyceae  Xanthophyceae | *Chlorellidium tetrabotrys*  *Heterococcus pleurococcoides* | AJ580949  AJ579335 |
| Xanthophyceae | *Bumilleriopsis peterseniana* | AJ579331 |
| Xanthophyceae | *Bumilleriopsis filiformis* | AF083398 |
| Xanthophyceae | *Botrydium stoloniferum* | U41648 |
| Xanthophyceae | *Tribonema intermixtum* | AF083397 |
| Xanthophyceae | *Mischococcus sphaerocephalus* | AF083400 |
| Xanthophyceae | *Pseudopleurochloris antarctica* | AF109729 |
| Xanthophyceae | *Sphaerosorus composita* | AJ579333 |
| Xanthophyceae | *Botrydiopsis intercedens* | U41647 |
| Xanthophyceae | *Botryochloris sp* | AJ579341 |
| Xanthophyceae | *Botrydiopsis alpina* | AJ579343 |
| Xanthophyceae | *Pleurochloris meiringensis* | AF109728 |
| Raphidophyceae | *Haramonas dimorpha* | AB365025 |
| Raphidophyceae | *Chattonella subsalsa* | AY788942 |
| Raphidophyceae | *Heterosigma akashiwo* | AB217869 |
| Raphidophyceae | *Vacuolaria virescens* | U41651 |
| Raphidophyceae | *Fibrocapsa japonica* | AY788931 |
| Pelagophyceae | *Pelagococcus subviridis* | U14386 |
| Pelagophyceae | *Coccoid pelagophyte* | U40926 |
| Pelagophyceae | *Aureococcus anophagefferens* | AF119119 |
| Pelagophyceae | *Chrysocystis sp* | AB183669 |
| Pelagophyceae | *Sarcinochrysis marina* | U78033 |
| Pelagophyceae | *Pulvinaria sp* | U78032 |
| Eustigmatophyceae | *Eustigmatos magna* | U41051 |
| Eustigmatophyceae | *Vischeria helvetica* | AF045051 |
| Eustigmatophyceae | *Pseudocharaciopsis minuta* | U41052 |
| Eustigmatophyceae | *Monodus unipapilla* | AM490827 |
| Eustigmatophyceae | *Pseudotetraedriella kamillae* | EF044311 |
| Eustigmatophyceae | *Nannochloropsis limnetica* | DQ977726 |
| Eustigmatophyceae | *Nannochloropsis gaditana* | EF473733 |
| Pinguiophyceae | *Pinguiococcus pyrenoidosus* | AF438324 |
| Pinguiophyceae | *Phaeomonas parva* | AB042204 |
| Pinguiophyceae | *Pinguiochrysis pyriformis* | AF438326 |
| Dictyochophyceae | *Pteridomonas* danica (DEN) | AB081640 |
| Dictyochophyceae | *Pteridomonas* danica (JAP*)* | L37204 |
| Dictyochophyceae | *Apedinella radians* | U14384 |
| Dictyochophyceae | *Pedinella sp* | AB081517 |
| Dictyochophyceae | *Rhizochromulina marina* | U14388 |
| Dictyochophyceae | *Florenciella parvula* | AY254857 |
| Dictyochophyceae | *Dictyocha speculum* | U14385 |
| Dictyochophyceae | *Chattonella verruculosa* | AB217630 |
| Oomycota | *Achlya apiculata* | AJ238656 |
| Oomycota | *Leptolegnia chapmanii* | AJ238660 |
| Oomycota | *Apodachlya brachynema* | AJ238663 |
| Synchromophyceae | *Synchroma grande* | DQ788730 |
| Chrysophyceae-Synurophyceae | *Mallomonas insignis* | EF165118 |
| Chrysophyceae | *Mallomonas splendens* | EF165147 |
| Chrysophyceae | *Synura uvella* | U73222 |
| Chrysophyceae | *Synura mammillosa* | U73220 |
| Chrysophyceae | *Tessellaria volvocina* | EF165119 |
| Chrysophyceae | *Paraphysomonas imperforata* | AF109324 |
| Chrysophyceae | *Monas sp* | AB168053 |
| Chrysophyceae | *Phaeoplaca thallosa* | AF123296 |
| Chrysophyceae | *Chrysosphaerella sp* | EF185316 |
| Chrysophyceae | *Chrysocapsa vernalis* | EF165105 |
| Chrysophyceae | *Chromophyton rosanoffii* | EF165107 |
| Chrysophyceae | *Chrysonebula flava* | EF165104 |
| Chrysophyceae | *Chrysochaete britannica* | AF123284 |
| Chrysophyceae | *Poterioochromonas malhamensis* | AB023070 |
| Chrysophyceae | *Spumella obliqua* | AJ236860 |
| Chrysophyceae | *Epipyxis pulchra* | AF123298 |
| Chrysophyceae | *Chrysamoeba mikrokonta* | AF123287 |
| Labyrinthulida | *Schizochytrium minutum* | AB022108 |
| Haptophyta | *Calcidiscus leptoporus* | AJ544116 |
| Haptophyta | *Umbilicosphaera foliosa* | AJ544119 |
| Haptophyta | *Pleurochrysis carterae* | AJ246263 |
| Haptophyta | *Phaeocystis cordata* | AF163147 |
| Haptophyta | *Prymnesium patelliferum* | L34670 |
| Haptophyta | *Emiliania huxleyi* | M87327 |
| Haptophyta | *Pavlova salina* | AF102987 |
| Haptophyta | *Coccolithus pelagicus* | AJ246261 |
| Haptophyta | *Cruciplaccolithus neohelis* | AJ246262 |
| Haptophyta | *Scyphosphaera apsteinii* | AM490984 |
| Haptophyta | *Helicosphaera carteri* | AM490983 |
| Haptophyta | *Chrysochromulina rotalis* | AM491025 |
| Alveolata | *Alexandrium tamarense* | AF022191 |
| Alveolata | *Alexandrium cohorticula* | AF113935 |
| Alveolata | *Ceratium fusus* | AF022153 |
| Alveolata | *Ceratium tenue* | AF022192 |
| Alveolata | *Gonyaulax spinifera* | AF022155 |
| Alveolata | *Peridinium bipes* | AF231805 |
| Alveolata | *Peridinium willei* | AF274272 |
| Alveolata | *Perkinsus sp* | L07375 |
| Alveolata | *Pyrocystis lunula* | AF274274 |
| Alveolata | *Noctiluca scintillans* | AF022200 |
| Uncertain classification | *Antarctosaccion applanatum* | AJ295822 |
| Uncertain classification | *Botrydiopsis pyrenoidosa* | AJ579337 |
| Viridiplantae | *Zygnema sp* | AF497796 |
| Viridiplantae | *Prasinococcus sp* | AF203400 |
| Rhodophyta | *Bangia fuscopurpurea* | AF169336 |
| Rhodophyta | *Glaucosphaera vacuolata* | AB045583 |
